# Effects of astaxanthin in mice acutely infected with *Trypanosoma cruzi*

**DOI:** 10.1051/parasite/2017018

**Published:** 2017-05-31

**Authors:** José María Eloy Contreras-Ortiz, Alberto Barbabosa-Pliego, Rigoberto Oros-Pantoja, José Esteban Aparicio-Burgos, José Antonio Zepeda-Escobar, Wael Hegazy Hassan-Moustafa, Laucel Ochoa-García, María Uxúa Alonso-Fresan, Esvieta Tenorio Borroto, Juan Carlos Vázquez-Chagoyán

**Affiliations:** 1 Centro de Investigación y Estudios Avanzados en Salud Animal, Facultad de Medicina Veterinaria y Zootecnia (FMVZ), Universidad Autónoma del Estado de México (UAEM) Kilómetro 15.5 Carretera Panamericana Toluca-Atlacomulco C.P. 50200 Toluca Estado de México; 2 Facultad de Medicina, Universidad Autónoma del Estado de México, Avenida Paseo Tollocan S/N, Moderna de la Cruz C.P. 50180 Toluca de Lerdo Estado de México; 3 Escuela Superior de Apan de la Universidad Autónoma del Estado de Hidalgo. Carr. Apan-Calpulalpan Km. 8, Chimalpa, Tlalayote S/N, Colonia Chimalpa Apan Hidalgo México; 4 Hospital Veterinario de Pequeñas Especies, Facultad de Medicina Veterinaria y Zootecnia (FMVZ), Universidad Autónoma del Estado de México, Jesús Carranza No. 203, Universidad 50130 Toluca de Lerdo México

**Keywords:** Astaxanthin, Chagas disease, *Trypanosoma cruzi*, Oxidative stress, Nifurtimox

## Abstract

During *Trypanosoma cruzi* infection, oxidative stress is considered a contributing factor for dilated cardiomyopathy development. In this study, the effects of astaxanthin (ASTX) were evaluated as an alternative drug treatment for Chagas disease in a mouse model during the acute infection phase, given its anti-inflammatory, immunomodulating, and anti-oxidative properties. ASTX was tested *in vitro* in parasites grown axenically and in co-culture with Vero cells. *In vivo* tests were performed in BALB/c mice (4–6 weeks old) infected with *Trypanosoma cruzi* and supplemented with ASTX (10 mg/kg/day) and/or nifurtimox (NFMX; 100 mg/kg/day). Results show that ASTX has some detrimental effects on axenically cultured parasites, but not when cultured with mammalian cell monolayers. *In vivo*, ASTX did not have any therapeutic value against acute *Trypanosoma cruzi* infection, used either alone or in combination with NFMX. Infected animals treated with NFMX or ASTX/NFMX survived the experimental period (60 days), while infected animals treated only with ASTX died before day 30 post-infection. ASTX did not show any effect on the control of parasitemia; however, it was associated with an increment in focal heart lymphoplasmacytic infiltration, a reduced number of amastigote nests in cardiac tissue, and less hyperplasic spleen follicles when compared to control groups. Unexpectedly, ASTX showed a negative effect in infected animals co-treated with NFMX. An increment in parasitemia duration was observed, possibly due to ASTX blocking of free radicals, an anti-parasitic mechanism of NFMX. In conclusion, astaxanthin is not recommended during the acute phase of Chagas disease, either alone or in combination with nifurtimox.

## Abstract (fr)

Pendant l’infection par *Trypanosoma cruzi*, le stress oxydatif est considéré comme un facteur contribuant au développement de la cardiomyopathie dilatée. Dans cette étude, les effets de l’astaxanthine (ASTX) ont été évalués comme un traitement médicamenteux alternatif pour la maladie de Chagas chez un modèle de souris pendant la phase d’infection aiguë, compte tenu de ses propriétés anti-inflammatoires, immunomodulantes et anti-oxydantes. L’ASTX a été testée *in vitro* sur des parasites cultivés axéniquement et en co-culture avec des cellules Vero. Des tests *in vivo* ont été effectués chez des souris BALB/c (âgées de 4-6 semaines) infectées par *T. cruzi* et traitées par ASTX (10 mg/kg/jour) et/ou nifurtimox (NFMX, 100 mg/kg/jour). Les résultats montrent que l’ASTX a des effets néfastes sur les parasites cultivés axéniquement, mais pas lorsqu’ils sont cultivés avec des monocouches de cellules de mammifères. *In vivo*, l’ASTX n’a eu aucune valeur thérapeutique contre l’infection aiguë par *T. cruzi*, utilisée seule ou en association avec NFMX. Les animaux infectés traités par NFMX ou ASTX/NFMX ont survécu à la période expérimentale (60 jours), tandis que les animaux infectés traités uniquement avec ASTX sont morts avant le 30ème jour après l’infection. L’ASTX n’a montré aucun effet sur le contrôle de la parasitémie; cependant, elle a été associée à une augmentation de l’infiltration focale lymphoplasmocytaire du cœur, un nombre réduit de nids d’amastigotes dans le tissu cardiaque et à des follicules de la rate moins hyperplasiques par rapport aux groupes témoins. De manière inattendue, l’ASTX a montré un effet négatif chez les animaux infectés co-traités avec NFMX. Une augmentation de la durée de la parasitémie a été observée, probablement due au blocage par l’ASTX des radicaux libres, un mécanisme antiparasitaire du NFMX. En conclusion, l’astaxanthine n’est pas recommandée pendant la phase aiguë de la maladie de Chagas, seule ou en association avec le nifurtimox.

## Introduction

Chagas disease is a zoonotic health concern in Latin America caused by *Trypanosoma cruzi*, with an estimated 6–7 million people infected. The infection is not limited to vectorial transmission, since it can be transmitted through blood transfusion or organ or tissue transplantation, and many cases of non-vectorial transmission have been reported in non-endemic areas [52].

The drugs available for the treatment of *Trypanosoma cruzi* infection in institutional health systems in Latin America are nifurtimox and benznidazole. However, these drugs have limited therapeutic value since they are effective only during the acute stages of the disease, and because these drugs may induce severe side effects in people undergoing long-term treatment [10, 50]. Furthermore, resistance to NFMX and benznidazole has been reported in parasites of different genotypes in endemic zones [9]. These therapeutic drawbacks leave people of all ages at risk [46, 47], and therefore, new strategies should be studied if an effective treatment is to be found.

During the acute phase of Chagas disease, an excessive production of free radicals in the heart has been correlated with irreversible oxidative stress (OS)-induced cardiomyocyte damage. Recent studies that analyzed the condition of the heart in Chagas disease have suggested that factors other than myocardial parasitism and autoimmune aggression are involved. It is unclear whether the tissue destruction is caused directly by factors related to the parasite, or indirectly by an immuno-inflammatory response amplified by the systemic overgeneration of reactive oxygen species (ROS) and reactive nitrogen species (RNS) [14, 6, 53]. Chagasic cardiomyopathy develops in 30–40% of chronically infected people. Cardiomyopathy may progress to cardiac insufficiency and sudden death because of progressive damage to cardiomyocytes and the ventricular intertruncal plexus [23].

Several studies in chronic chagasic patients suggest that the use of antioxidants, such as vitamin E and C, decreases free radical levels and the OS associated with the disease [30, 42], protecting the myocardium and preventing the progression of chagasic cardiomyopathy into more severe syndromes [51]. Astaxanthin (ASTX), a reddish carotenoid that belongs to the xanthophyll class, is a potent antioxidant naturally found in several sea animals (*Haematococcus pluvialis*) and plant species [18, 22]. It has anti-inflammatory [25] and immunomodulatory properties [12], which can stabilize free radicals and decrease oxidative stress damage, protecting biologically important molecules. Studies have shown that ASTX counteracts OS caused by some heart diseases, preventing tissue damage caused by cell oxidation and contributing to a healthier myocardium [17, 34]. Here, we evaluated the effects of ASTX supplementation during the acute phase of Chagas disease in an induced infection with a pathogenic strain (*Ninoa*) of *Trypanosoma cruzi* in BALB/c mice.

## Materials and methods

### Ethics

Mice were kept, fed, and reared under standard conditions (18–23 °C, 50–60% relative humidity), according to the guidelines of the Bioethics Committee of the FMVZ-UAEM, the Official Mexican Standard regarding technical specifications for the care and use of laboratory animals (NOM-062-ZOO-1995) [37], and the standards of the National Academy of Science [35].

### Parasite culture

Trypomastigotes of *Trypanosoma cruzi*, *Ninoa* strain (TCI) (kindly donated by Dr. Pedro Reyes from the *Instituto Nacional de Cardiología* “Ignacio Chávez”), were used to infect Vero cell monolayers, which were maintained in Dulbecco’s minimal essential medium (DMEM [Gibco Laboratories, USA]), supplemented with 2% fetal bovine serum (FBS [Gibco Laboratories, USA]) and 1% penicillin-streptomycin (Gibco Laboratories, USA), under controlled conditions (37 °C, 5% CO_2_, and saturated humidity) [31].

### Parasite harvest from cell culture

Parasites were cultured for 1–2 weeks on Vero cell monolayers, when they started to break out from the infected cells. The medium with free-swimming parasites was then collected in 15 mL sterile conical tubes and centrifuged at 2700 rpm for 7 min. The supernatant was discarded and the pellet was resuspended in 1 mL of DMEM (Gibco Laboratories, USA). Parasites were counted using a hemocytometer, and the number of parasites was adjusted to the specific needs of each assay (*in vitr*o or *in vivo*).

### Astaxanthin preparation for *in vitro* assays

In order to purify astaxanthin from the commercial preparation for the *in vitro* assay, one gram of microencapsulated astaxanthin (AstaPure^®^, Algatechnologies, Israel) was ground in a sterile mortar, placed in a 15 mL sterile conical tube, and suspended in 6 mL of extraction solution (petroleum ether:acetone:water, [15:75:10]) [33]. The suspension was mixed by inversion several times and gently vortexed for 15 min. The tube was centrifuged at 7500 rpm for 10 min at 4 °C, and the supernatant collected in a fresh sterile 15 mL tube. The solvents were evaporated at 40 °C for 12 h in dark conditions and the astaxanthin was resuspended in 2 mL of DMEM-dimethyl sulfoxide (DMSO [Sigma-Aldrich, USA]) (99.7/0.3% V/V solution). This suspension was gently vortexed for 10 min, and then filtered using an acrodisc syringe filter (0.22 μm) in a 1.5 mL sterile tube and kept at 4 °C until use. The ASTX concentration was determined in a 96-well plate using a *β*-carotene (Sigma-Aldrich, USA) standard curve and read at 450 nm in a spectrophotometer (BioTek, USA). A simple linear regression was used to determine ASTX concentrations in μg/μL.

### Astaxanthin *in vitro* toxicity assay for *T*. *cruzi* and Vero cells

Trypomastigotes (5 × 10^5^/well) or Vero cells (2 × 10^4^/well) were cultured in a 96-well plate (Sarstedt, USA) in supplemented DMEM (2% FBS, penicillin 10,000 units/mL, and streptomycin 10,000 μg/mL) and astaxanthin at 1, 5, 10, 20, or 30 μg/100 μL. The assay was performed in triplicate with the following controls: a) C-T (untreated trypomastigotes), b) C-V (untreated Vero cells), c) DMEM/DMSO in a proportion equivalent to the amount of DMSO used in the highest ASTX dose (99.7/0.33% V/V, respectively) (this control was necessary since ASTX and NFMX were solubilized in this solvent), and d) nifurtimox (400 μg/100 μL) (Lampit^®^, Bayer). NFMX was prepared as previously described by Rolón et al. [43]. One tablet of the commercial presentation of NFMX (120 mg) was ground in a sterile mortar and resuspended in 1 mL of DMSO. The final DMSO concentration in the culture media never exceeded 0.3% in a V/V solution. Plates were incubated for 24 h in controlled conditions (37 °C, 5% CO_2_, and saturated humidity). After treatment, the viability of parasites and cells was estimated using MTS (3-[4,5,dimethylthiazol-2-yl]-5-[3-carboxymethoxy-phenyl]-2-[4-sulfophenyl]-2H-tetrazolium, inner salt) from CellTiter 96 kit^®^ Aqueous One Solution (Promega, USA), following the manufacturer’s instructions. The metabolic activity of parasites and cells over MTS was estimated by colorimetry at 490 nm wavelength. In this assay, the higher the optical density (OD) values, the higher the cell viability.

### Morphologic evaluation of changes induced by ASTX on Vero cells and *T. cruzi* co-cultures

Vero cells (5 × 10^3^/well) were seeded and cultured for 24 h as previously described and then infected with trypomastigotes (10 parasites/cell) [13]. Once intracellular parasites were observed (about 96 h after infection), the old medium was replaced with fresh supplemented DMEM with different ASTX doses (1, 5, 10, 20, or 30 μg/100 μL). As a control, co-cultures were kept with NFMX (400 μg/100 μL) or with no ASTX or NFMX supplementation. After 24 h of incubation, microscopic morphological changes in the co-culture, such as loss of normal shape of *T. cruzi* infected Vero cell, changes of normal parasite shape or motility, and variations in the presence of intra- or extra-cellular parasites were evaluated by a trained technician. Additionally, parasite viability was evaluated by Trypan blue stain assay [2].

### Animals and challenge

BALB/c female mice (*N* = 48), 4–6 weeks old, were distributed in eight groups (*n* = 6): G1 (Tc); G2 (Tc/ASTX); G3 (Tc/ASTX/NFMX); G4 (Tc/NFMX) and four non-infected controls: G5 (saline solution); G6 (NFMX); G7 (ASTX/NFMX); and G8 (ASTX). Animals from groups G1 to G4 were infected intraperitoneally with 10 trypomastigotes each. Specimens were clinically evaluated on a daily basis; any animal health changes, such as weight loss, hirsutism, morbidity, lameness, or any other behavioral changes, were recorded. We decided to use ASTX during the acute phase of infection in BALB/c mice because there are no previous reports on the use of antioxidants at this stage of infection and because in *in vitro* experiments in our laboratory, ASTX had some antiparasitic effect. We also decided to test ASTX as an antiparasitic agent during an early stage of infection in BALB/c mice because this mouse strain is susceptible to infection with *Ninoa* strain of *T. cruzi* with a predictable outcome and the parasitemia is easily detected. Therefore, during the acute phase of infection, the level of parasitemia was used as an indicator of disease development [15], providing an easy-to-evaluate parameter, to determine the possible effects of ASTX on the infection while animals were alive.

### Parasitemia

Parasitemia was analyzed for each mouse, by fresh blood smear test. Samples were collected twice a week starting on day 5, until day 60 post-infection, or when parasitemia was undetectable microscopically in fresh blood preparations. Sampling was performed according to Brener [7] with slight modifications. Briefly, a small cut was performed on the tip of the tail of the mouse, blood (4 μL) was collected with a micropipette, placed on a glass slide, and covered with a coverslip (18 × 18 mm). Samples were observed under light microscopy at 400×. Parasites in 100 fields were counted, and the number of parasites/μL was estimated with standard protocols [28, 45].

### ASTX supplementation and NFMX treatments for *in vivo* assays

From day 12 onwards, ASTX and/or NFMX (Lampit^®^, Bayer) were administered according to the animals’ treatment group (Table 1). ASTX was prepared from 400 mg beadlets of AstaPure^®^. Beadlets were ground in a sterile mortar in aseptic conditions and resuspended and homogenized in 3 mL of a 20% (V/V) sterile solution of Tween-20/distilled water [36] for a final volume of 3.1 mL. ASTX supplementation (60 μL of ASTX preparation, equivalent to 10 mg/kg/day of pure ASTX) was orally administered with a micropipette until day 60 post-infection. This concentration has exhibited immunomodulatory and anti-inflammatory effects in mice and other species, including humans [24, 27, 34, 40]. NFMX was prepared in aseptic conditions by grinding one tablet containing 120 mg of NFMX (Lampit^®^, Bayer) in a mortar and resuspending it in 1 mL of sterile distilled water [11]. This solution was administered orally at a single daily dose of 100 mg/kg/day [8] (Table 1) in a 60 μL volume. Treatment was carried out until the day when parasitemia could no longer be detected through fresh blood preparations, as described above [6, 28, 45].

**Table 1.** Description of treatments used in *in vivo* experiments.

Mice groups (*n* = 6)	*T. cruzi* Ninoa strain (Infection dose)	Astaxanthin dose (mg/kg/day)	Nifurtimox dose (mg/kg/day)
G1 (Tc)	10 parasites		
G2 (Tc/ASTX)	10 parasites	10	
G3 (Tc/ASTX/NFMX)	10 parasites	10	100
G4 (Tc/NFMX)	10 parasites		100
G5 (saline solution)			
G6 (NFMX)			100
G7 (AST/NFMX)		10	100
G8 (ASTX)		10	

Tc: Challenge with *T. cruzi* (positive control); ASTX: Astaxanthin; NFMX: Nifurtimox; G5–G8 (controls). Six BALB/c mice were used per group.

### Animal sacrifice and tissue sampling

Heart and spleen tissues were collected from mice after they died from infection or when they were euthanized. Mice were sacrificed either because they were very ill or on day 60 after infection. Euthanasia was performed by cervical dislocation following protocols established by *Norma Oficial Mexicana* (NOM-033-ZOO-1999) [38], the Bioethics Committee from UAEM-FMVZ, and from the Council for International Organizations of Medical Sciences [35]. Blood samples were taken directly from the heart to obtain sera on the day of sacrifice and tissues were fixed in 10% formaldehyde for histopathological studies.

### Histopathological study

Tissues were fixed in 10% formaldehyde for 24 h, dehydrated in absolute ethanol, and included in paraffin. Tissue sections (5 μm) were prepared and stained with hematoxylin-eosin and observed under light microscope (Carl Zeiss Axiostar, USA). Images were recorded with a Tucsen 5 MP camera (Tucsen, China) with the Image-Pro Plus 7 software. Tissue samples were studied microscopically at 400× magnification to assess parasite burden (amastigote nests observed in 100 random fields). The severity of inflammation was estimated by the severity of lymphocyte infiltration in the tissue, in 400 random fields, using the scale proposed by Barbabosa-Pliego et al. [5]: (−), none; (+), light; (++), moderate; and (+++), severe.

### Malondialdehyde (MDA) assay

Malondialdehyde levels were determined in sera following the instructions of an OxiSelect^TM^ MDA Adduct ELISA Kit (Cell Biolabs, USA). Standards and samples were incubated in a 96-well plate for 2 h, at 37 °C. The MDA-protein adducts present in the sample and in the standards were probed with an anti-MDA antibody followed by the HRP-conjugated secondary antibody, revealed with 3,3′,5,5′-tetramethylbenzidine (TMB) and read by spectrophotometry at 450 nm. The MDA-protein adducts content in each sample was determined by comparison with a standard curve that was prepared from predetermined MDA-BSA standard [16]. A simple linear regression was used to determine the MDA concentration in pmol/mL.

### Statistical analysis

Analysis of variance (ANOVA) was used to analyze results from the *in vitro* viability assay, parasitemia, and MDA. Mean differences for all assays were assessed by a Tukey test, except for parasitemia where a Bartlett’s test was used. Statistical analyses were conducted with the GraphPad Prism 5.0 software package (GraphPad Software Inc., USA). Differences were considered significant at *p* < 0.05.

## Results

### *In vitro Trypanosoma cruzi* and Vero cell viability after exposure to ASTX

**Figure 1.**
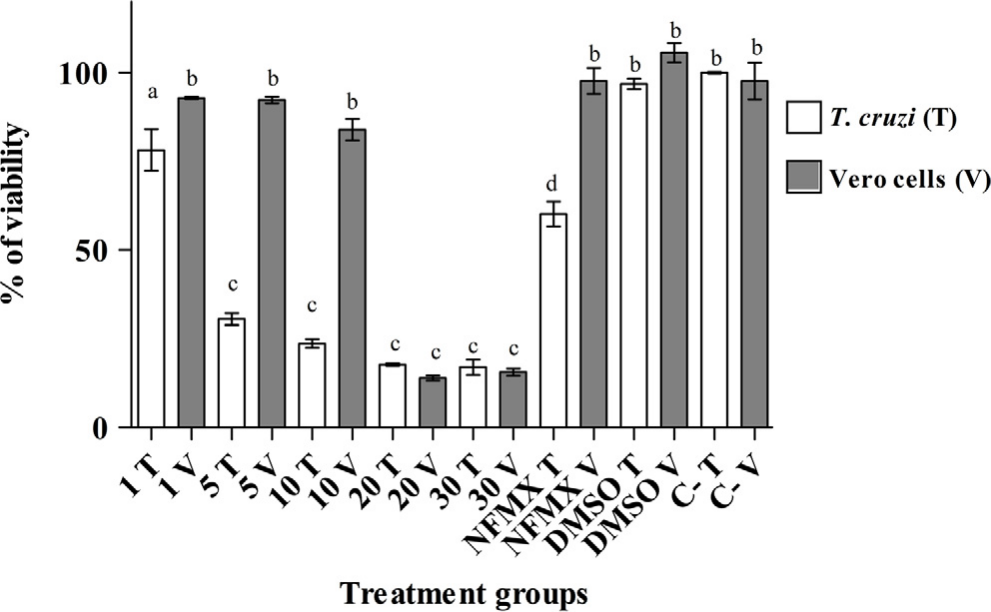
*Trypanosoma cruzi* trypomastigote and Vero cell survival after treatment with five different doses of astaxanthin (ASTX, 1, 5, 10, 20, or 30 μg/100 μL). Nifurtimox (NFMX, 400 μg/100 μL), dimethyl sulfoxide (DMSO, 0.03%), or no treatment was used as control for the MTS viability assay. Samples were evaluated after 24 h of treatment. Each bar represents the absorbance mean value ± *SD*. Differences (*p* < 0.05) among groups, according to Tukey’s test, are indicated with characters on top of treatment bars.

Trypomastigote and Vero cell viability was evaluated 24 h after treatment. Figure 1 shows parasite and Vero cell survival after treatment, either with ASTX (1, 5, 10, 20, or 30 μg/100 μL), NFMX (400 μg/100 μL), DMSO (0.33% V/V), or untreated (C-). Parasite viability was progressively affected (*p* < 0.05) as ASTX doses were increased; from nearly 100% parasite survival (with no ASTX) down to 18% survival at the higher doses (20–30 μg/100 μL) of ASTX. Vero cell viability was only significantly compromised at 20 and 30 μg/100 μL ASXT doses (*p* < 0.05). NFMX affected parasite viability (*p* < 0.05) at a 400 μg/100 μL dose and did not compromise Vero cell survival. No apparent viability of parasites or Vero cells was affected after the use of DMSO (0.3% V/V).

Parasites were not affected by ASTX (1–20 μg/100 μL) when evaluated in co-culture with Vero cells, unlike the results observed in axenic culture (Table 2). These results call into question whether the effects of ASTX would be detrimental or not to the parasite in an *in vivo* model, and therefore we decided to continue testing ASTX as a therapeutic treatment in an experimental animal model.

**Table 2.** Effects of ASTX in *T. cruzi* infected Vero cell culture (24 h post-treatment).

ASTX or NFMX doses	1 μg		5 μg		10 μg		20 μg		30 μg		NFMX 400 μg	
Parameters/Cell	Tc	Vc	Tc	Vc	Tc	Vc	Tc	Vc	Tc	Vc	Tc	Vc
IP	+		+		+		+		−		−	
EP	+		+		+		+		−		−	
Motility	+		+		+		+		−			
Viability	+	+	+	+	+	+	+	+	−	−		+
Loss of cellular form	−	−	−	−	−	−	−	−	+	+		+
Integrity of the cell membrane	+	+	+	+	+	+	+	+	−	−		+

ASTX dose (astaxanthin, 1–40 μg); NFMX (nifurtimox 400 μg); IP (intracellular parasite); EP (extracellular parasite); + (presence); − (absence); Tc (*Trypanosoma cruzi*); Vc (Vero cell). Integrity of the cell membrane was evaluated through Trypan Blue assay [28, 45].

### Parasitemia in BALB/c mice infected with *T. cruzi*

**Figure 2.**
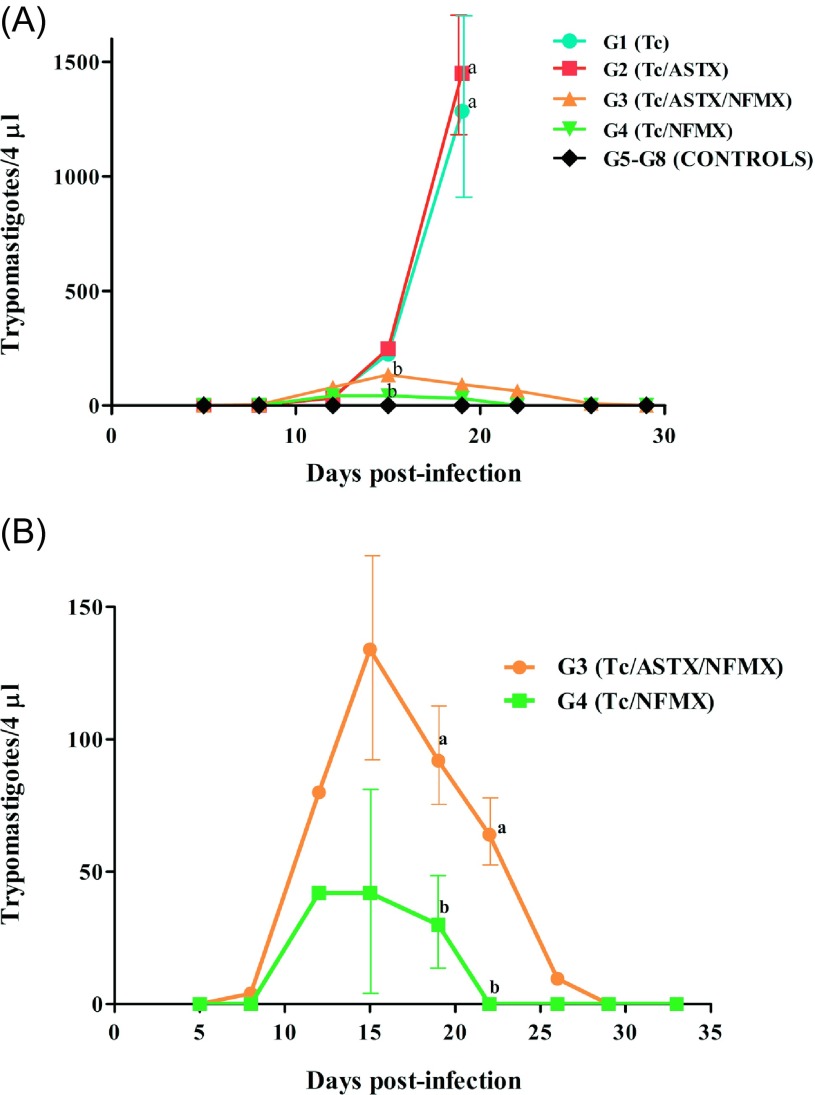
(A): Blood parasitemia observed in mice acutely infected with *Trypanosoma cruzi* (Tc) and treated with astaxanthin (ASTX) and/or nifurtimox (NFMX). Controls included infected animals with no treatment at all (G1), or animals treated with ASTX and/or NFMX without *T. cruzi* infection (G5–G8). Blood samples (4 μL) were collected and microscopically analyzed every other day from days 5 to 30 post-infection. Mean number of parasites ± *SD*, (B): Detail of parasitemia for groups G3 (Tc/ASTX/NFMX) and G4 (Tc/NFMX). Different characters above lines show statistical differences (*p* < 0.05) among treatments within the same day of sampling according to Tukey’s test.

Experimental groups showed differences in the number of blood trypomastigotes (Fig. 2). Challenged groups G1 (Tc) and G2 (Tc/ASTX) showed the highest parasitemia and did not survive beyond day 23 post-infection. It is worth mentioning that ASTX supplementation on its own, in infected animals, did not show any survival advantage over the control group. Challenged groups G3 and G4, treated with ASTX/NFMX or just NFMX, respectively, developed low levels of parasitemia. This was controlled by days 28 and 22 post-infection, respectively (Figs. 2A and 2B). Parasitemia levels in groups G3 (33 ± 12.7 parasites/μL) and G4 (10 ± 5 parasites/μL) were statistically different (*p* < 0.05) from those found in animals in groups G1 (321 ± 138.2 parasites/μL) and G2 (362 ± 156.2 parasites/μL) around day 20 post-infection. All non-infected animals were in good health until the day of sacrifice (day 60 post-infection).

### Anatomopathologic findings

#### Heart

The size of the heart in all experimental groups (G1–G8) did not show differences. Hearts were measured in sagittal position and average length was 0.79 ± 0.036 cm. No apparent morphological changes were found macroscopically.

#### Spleen

**Figure 3.**
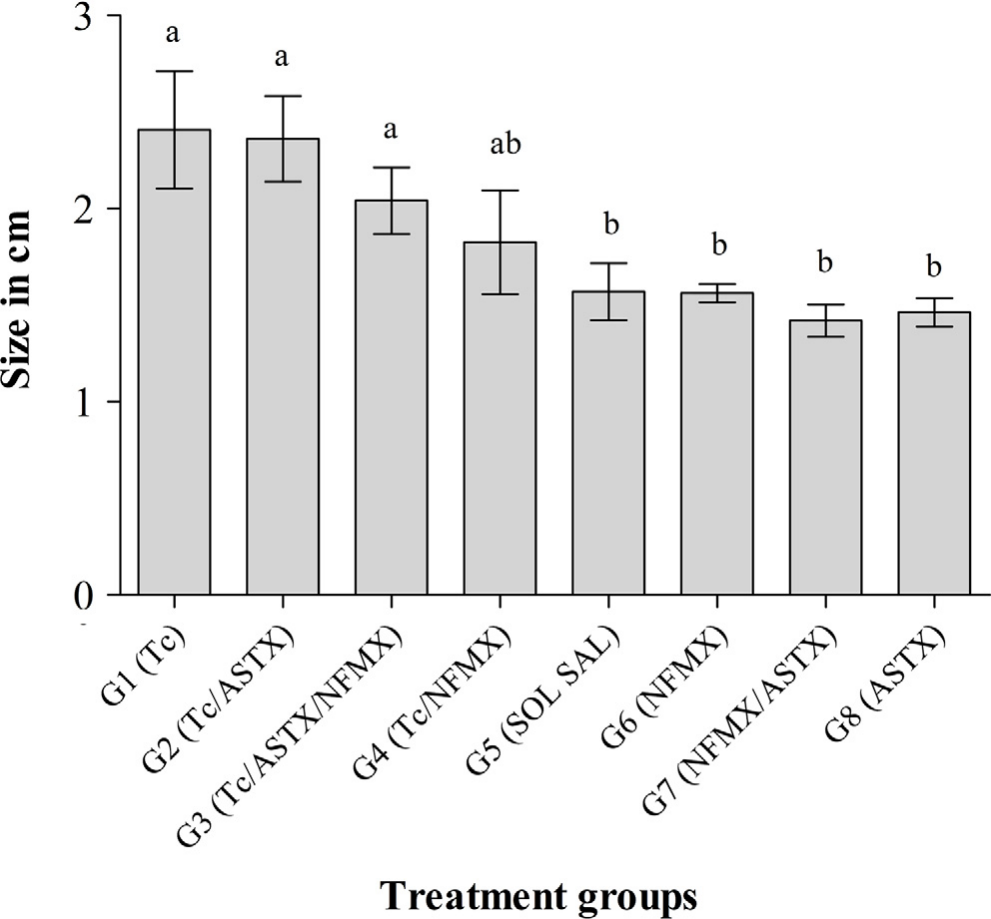
Spleen size of animals experimentally infected with *Trypanosoma cruzi.* Spleens were collected immediately after the animal died due to infection or on the day of sacrifice (60 days post-infection). Each bar represents the mean size value ± *SD*. Statistical differences (*p* < 0.05) among groups are shown with different characters above the bars according to Tukey’s test.

Spleens were clearly enlarged in all *T. cruzi-*challenged groups (G1–G4), where splenomegaly was observed (Fig. 3). The average size of the spleen was 2.4 ± 0.26 cm for animals from groups G1 (Tc) and G2 (Tc/ASTX), and 2 ± 0.17 and 1.8 ± 0.26 cm in groups G3 and G4, respectively. All control groups (G6–G8) had an average spleen size of 1.5 ± 0.08 cm, similar in size and appearance to mice treated with saline solution (G5), which was considered normal.

### Histopathologic findings

#### Heart

**Figure 4.**
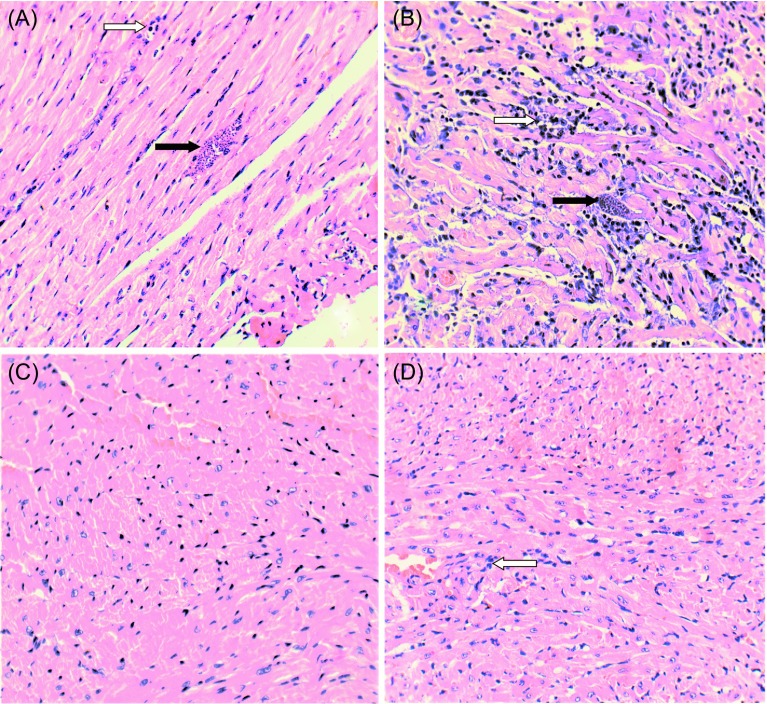
Histological analysis of heart tissue sections from acutely *Trypanosoma cruzi* infected mice, treated with astaxanthin and/or nifurtimox. Heart tissue sections from the left ventricle were processed on the day animals died (either due to infection or when euthanized on day 60 post-infection). Tissue sections (5 μm) were stained with hematoxylin-eosin. Representative micrographs are shown for mice from the following groups: (A) G1 (Tc); (B) G2 (Tc/ASTX); (C) G5 (saline solution); (D) G8 (ASTX). The micrograph from G5 could represent all groups from G3 to G7; all of them were considered histologically normal. Black arrow, amastigote nests; white arrow, lymphoplasmacytic infiltrate. (400× amplification).

Left ventricle sections displayed differences among treatment groups. Group G1 (*T. cruzi*) had the largest number of amastigote nests (*n* = 35 ± 3.9), and the myocardium displayed diffuse inflammatory infiltrates, represented mainly by lymphoplasmacytes (Fig. 4A). In G2 (Tc/ASXT) the number of amastigotes (*n* = 24 ± 3.05) was significantly lower (*p* < 0.05), but these animals had increased local inflammation and a higher number of necrotic cardiomyocytes (Fig. 4B). The G3 and G4 groups showed light focal lymphoplasmacytes infiltrate and necrotic cardiomyocytes, with no amastigote nests present. Non-infected control groups (G5–G8) were normal (Figs. 4C and 4D, Table 3).

**Table 3.** Myocardial histopathological abnormalities found in mice during the acute phase of *T. cruzi* experimental infection (60 days post-infection)

Parameters/group	G1	G2	G3	G4	G5	G6	G7	G8
Focal lymphoplasmacytes	−	++	+	+	−	−	−	−
Diffuse lymphoplasmacytes	+	−	−	−	−	−	−	−
Cardiomyocyte necrosis	+	++	+	+	−	−	−	−
Amastigote nests (mean ± *SD*)	35 ± (3.9)	24 ± (3.05)	0	0	0	0	0	0

Treatment groups: G1: *T. cruzi*; G2: *T. cruzi*/ASTX; G3: *T. cruzi*/ASTX/NFMX; G4: *T. cruzi*/NFMX; G5: saline solution; G6: NFMX; G7: ASTX/NFMX; G8: ASTX. Abnormality scale: −, none; +, light; ++, moderate; and +++, severe [5]; ±: standard deviation.

#### Spleen

**Figure 5.**
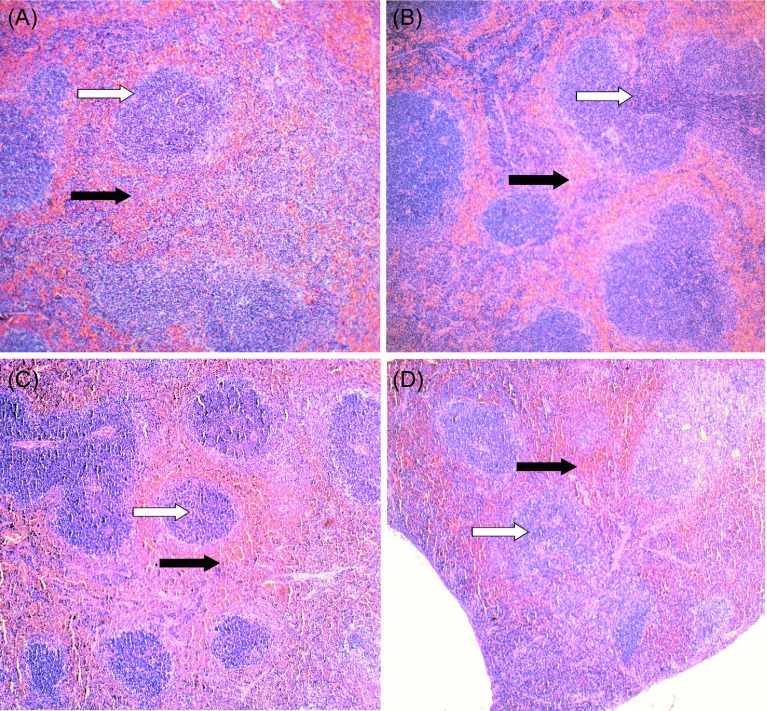
Histological analysis of spleen tissue sections in acutely *Trypanosoma cruzi* infected mice, treated with astaxanthin and/or nifurtimox. Spleen tissue sections (5 μm) were obtained at 60 days post-infection or at the time the animals died due to infection, and stained with hematoxylin-eosin. Representative micrographs of mice (BALB/c) from the following groups are shown: (A) G1 (Tc); (B) G2 (Tc/ASTX); (C) G5 (saline solution); (D) G8 (ASTX). The micrograph from G5 could represent all groups from G3 to G7; all of them were considered histologically normal. White arrow: lymphoid follicles; Black arrow: red pulp.

Morphological changes in the spleen were observed mainly as hyperplasia of lymphoid follicles and loss of characteristic shape. The G1 (Tc) group showed very diffuse and extended follicles with severe hyperplasia of lymphoid follicles (Fig. 5A). In animals from the G2 group (Tc/ASTX supplementation), slight hyperplasia of lymphoid follicles was observed (Fig. 5B). In groups G3 (Tc/ASTX/NFMX) and G4 (Tc/NFMX), as well as in control groups (G5–G8), lymphoid follicles appeared normal, with no pathological changes (Figs. 5C and 5D).

### Malondialdehyde (MDA) test

**Figure 6.**
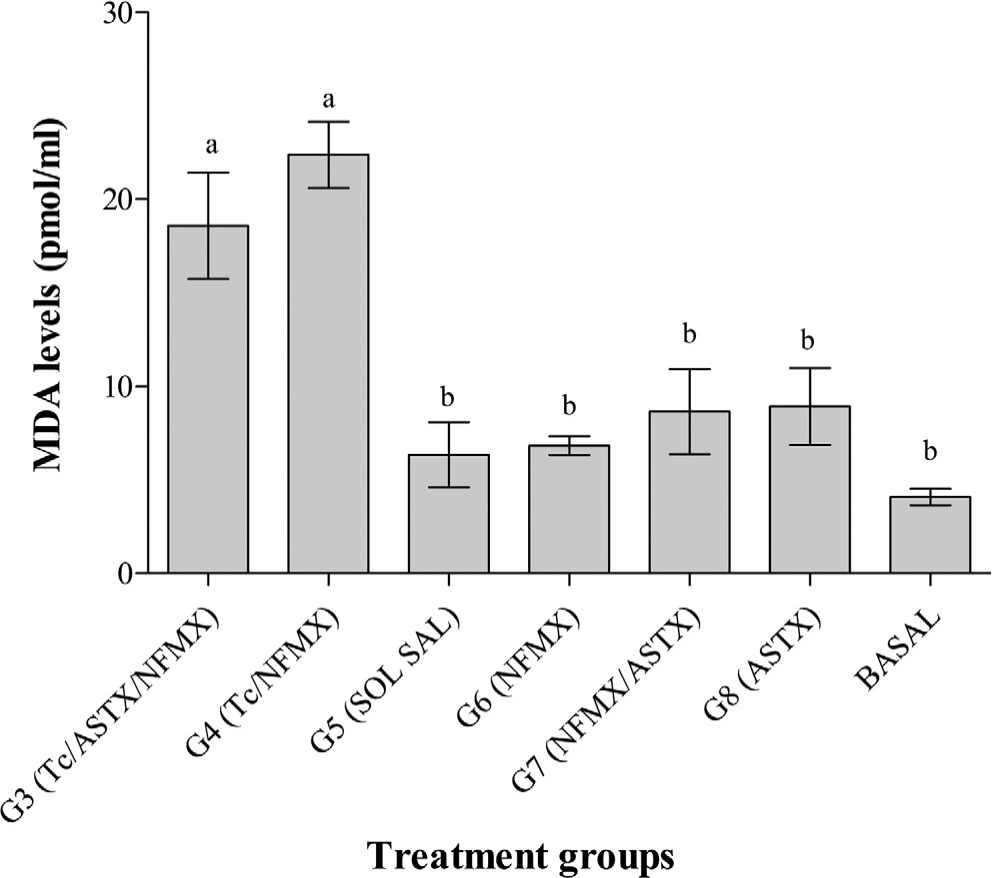
Malondialdehyde serum levels in animals after experimental *Trypanosoma cruzi* infection under treatments G3–G8 at the day of sacrifice (60 days post-infection). Each bar represents the mean pmol/mL value ± *SD*. Statistical differences (*p* < 0.05) among groups are shown with different characters above the bars. Groups G1 and G2 were not included because the mice died before day 30 post-infection.

It is important to note that groups G1 (Tc) and G2 (Tc/ASTX) were not incorporated in the assay because these animals did not survive the acute phase of the disease, and blood samples could not be collected. From the remaining groups, the highest levels of MDA in sera were found in animals from groups G3 (Tc/ASTX/NFMX) and G4 (Tc/NFMX) with 18.5 ± 2.8 pmol/mL and 22.2 ± 1.7 pmol/mL, respectively. Control groups, G5 (saline solution), G6 (NFMX), G7 (NFMX/ASTX), and G8 (ASTX), showed 6.3 ± 1.7, 6.8 ± 0.5, 8.7 ± 2.2, and 8.9 ± 2 pmol/mL of MDA, respectively, while the basal levels (mouse sera without manipulation and treatment) were 4.0 ± 0.4 pmol/mL (Fig. 6).

## Discussion

Several *in vitro* research studies have reported that the antioxidants found in some plants might have a detrimental effect on the viability of different parasites [1, 19] including Trypanosomatids [29, 32, 48, 49]. In our laboratory, initial *in vitro* results showed that ASTX induced *T. cruzi* trypomastigote death in a dose-dependent manner (Fig. 1). Therefore, we wanted to address the question of whether ASTX would be able to control an *in vivo T. cruzi* infection using a mouse model. Results did not support our hypothesis, since ASTX did not control *in vivo* parasitemia loads (Fig. 2A), and the infected animals treated only with the antioxidant (G2) died during the acute phase of infection, as occurred with infected animals with no treatment (G1 group). Furthermore, ASTX seemed to interfere with the efficacy of NFMX against the parasites *in vivo*, since parasitemias observed in animals from group G3 (Tc/ASTX/NFMX) were significantly higher (*p* < 0.05) and longer (*p* < 0.05), than parasitemias found in infected animals from group G4 treated only with NFMX (Fig. 2B). Therefore, also considering the results reported by Wen et al. [51], who found that PBN (phenyl-*α*-tert-butyl-nitrone), a synthetic antioxidant, used in Sprague Dawley rats infected with *T. cruzi*, did not decrease parasite load during the acute phase of infection, it could be concluded that the use of antioxidants is not indicated during this phase of Chagas disease. However, strong antioxidants, such as ASTX, could still be useful during the chronic phase of Chagas disease. This idea is supported by the findings of Maçao et al. [30] and Ribeiro et al. [42], who found that supplementation with vitamins E and C after the use of benznidazole for the treatment of Chagas disease in humans reduced oxidative stress, and contributed to minimizing the risk of chagasic cardiomyopathy in chronically infected patients. If we consider that ASTX is a stronger antioxidant than vitamins E and C, and additionally that it has anti-inflammatory and immunomodulatory properties [17, 34, 40], the question that remains to be answered is whether ASTX supplementation, after the administration of anti-chagasic agents such as benznidazole or nifurtimox during the chronic phase of Chagas disease, would be beneficial to improve chronic chagasic cardiomyopathy.

When comparing the histopathological appearance of the left ventricle from animals in groups G1 (Tc) and G2 (Tc/ASTX), it was observed that G2 animals had an increment in the number of focal lymphoplasmacytic infiltrations and necrotic cardiomyocytes, and a lower number of amastigote nests (Figs. 4A, 4B and Table 3). These differences suggest that ASTX had an immunomodulatory effect, which would promote the strong immune reaction observed, accompanied by a lower number of amastigote nests in cardiac tissue. It has been reported that the immunomodulatory properties of ASTX include the stimulated proliferation of T and B lymphocytes and NK cells, production of pro-inflammatory cytokines such as IL-1a and TNF-*α*, as well as promoting an increment in antibody production against various antigens [4, 12, 39, 40]. Therefore, it would be interesting to further study whether ASTX could be used as a therapeutic drug in Chagas disease, either in combination with an anti-*T. cruzi* non-oxidative stress-inducing drug or in combination with antiparasitic (prophylactic or therapeutic) vaccines.

Splenomegaly has been reported in animals and humans infected with *T. cruzi*. This reaction is related to host inflammatory responses to the parasitic infection [41], and reactive oxygen species (ROS) generated by neutrophils and macrophages in the spleen [3, 4, 44], which induce the expression of inflammatory genes that contribute to inflammation [26]. In the present study, animals from non-infected groups had an average spleen size of 1.5 cm with normal histology. In comparison, animals from all infected groups (G1–G4) showed splenomegaly. The average spleen size for groups G1 (Tc) and G2 (Tc/ASTX) (Fig. 3) was 2.3 cm, i.e. 53% larger in comparison with the non-infected control groups. These spleens displayed hyperplasic lymphoid follicles (Fig. 5A). Animals from group G3 (Tc/ASTX/NFMX) and G4 (Tc/NFMX) showed 33% (2.1 cm) and 20% (1.8 cm) larger spleens than normal animals, respectively (Fig. 3). This inflammatory response could be partially explained by the fact that, before infection was controlled by NFMX, there was a period when parasites proliferated in the animals and inflammation was triggered.

Oxidative stress is one of the main features of the immune system that is triggered during the development of chagasic cardiomyopathy [20]. Oxidative stress induced by *T. cruzi* infection in the myocardium can be studied through markers such as MDA [16]. Our results showed statistical differences between serum MDA from infected (G3 and G4 groups) and non-infected animals (G5–G8 groups) (Fig. 6). However, unlike what was expected, no differences were observed in non-infected animals among groups receiving NFMX, ASTX/NFMX, and ASTX or saline solution. This outcome is difficult to explain as NFMX was expected to increase MDA values and ASTX to reduce them. A possible explanation could be that the MDA assay used to detect OS was not sensitive enough to identify small differences, and that the effects of NFMX and ASTX on mouse physiology were not large enough to be detected. *T. cruzi* infection did induce OS and was detected by the MDA assay. However, no statistical differences were observed between serum MDA levels from groups G3 and G4. We had hypothesized that animals receiving ASTX would have lower levels of OS [17], but this could not be proven. This outcome could probably also be explained if we assume that the doses of ASTX used in this experiment were not sufficiently high to promote an antioxidant effect detectable by the MDA assay. As a whole, the findings of the present study do not support the idea that ASTX has a positive effect during an acute *T. cruzi* infection and the question that remains to be answered is whether ASTX could be used in chronic Chagas infections to possibly improve the results observed with other antioxidants, such as vitamins E and C or synthetic antioxidants such as PNB, in chronically infected chagasic human patients [30] and rats [51], considering that ASTX is a more active antioxidant than those previously described [21].

## Conclusions

The use of ASTX during the acute phase of *T. cruzi* infection is not recommended, whether alone or in combination with therapeutic drugs that induce oxidative stress, such as NFMX. However, the potential beneficial effects of ASTX if used in the chronic phase of Chagas disease, or in combination with non-OS–inducing antiparasitic drugs, or with prophylactic or therapeutic vaccines, remain to be studied.

## Conflict of interest

The authors declare that they have no conflict of interest.
